# Laparoscopic extraction of a urethral self-inflicted needle from pelvis in a boy: a case report

**DOI:** 10.3389/fped.2023.1207247

**Published:** 2023-06-23

**Authors:** Xiaoqing Wang, Xiangyu Wu, Wei Liu, Guoqiang Du, Yanze Wang, Rongde Wu, Feng Guo

**Affiliations:** Department of Pediatric Surgery, Shandong Provincial Hospital Affiliated to Shandong First Medical University, Jinan, China

**Keywords:** foreign body (FB), urethra, migration, laparoscopy, children, case report

## Abstract

**Introduction:**

Self-insertion of foreign bodies in the urethra is an infrequent occurrence in children, and their management aims to minimize urethral morbidity. Endoscopic removal presents a significant challenge, particularly in boys. Currently, there are few reports on laparoscopic management of urethral foreign bodies that have migrated to the pelvic cavity.

**Case description:**

An 11-year-old boy presented to the emergency department with complaints of increased frequency of micturition and dysuria. A sharp sewing needle was discovered lodged in the posterior urethra mucosa during cystoscopy. Attempts to remove the needle using an endoscopic grasping forceps were unsuccessful due to the forceps' weak biting power. During a digital rectal examination, the needle migrated into the pelvic region, wedged between the prostatic urethra and the rectal ampulla. After careful inspection of the peritoneal reflection over the fundus of the bladder, the needle was identified and successfully removed through laparoscopy without any complications. Psychiatric counseling was advised for this patient, who was in good condition during an 8-week follow-up.

**Conclusions:**

Our case demonstrates the first recorded use of laparoscopy to remove a self-inserted urethral needle that had migrated into the pelvic region, after failed attempts at endoscopic extraction. Future cases may benefit from considering laparoscopic interventions for similar circumstances.

## 1. Introduction

Urethral self-insertion of foreign bodies (FBs) in children are occasionally reported (1–6). The goal of management is to remove FBs with minimal or no urethral morbidity. However, endoscopic handling of urethral FBs presents a significant challenge, particularly in boys, due to the size of urethra (3). Most published literatures report treatment of FBs in the lower urinary tract through urethrocystoscopy or open cystotomy but few involve laparoscopic handling of urethral FBs that have migrated into pelvic cavity. Herein we present the first case of an 11-year-old boy with a self-inflicted intraurethral needle, which penetrated through the urethra during extraction and was retrieved laparoscopically from the anterior reflection of the peritoneum without complications.

## 2. Case description

**Figure 1:**
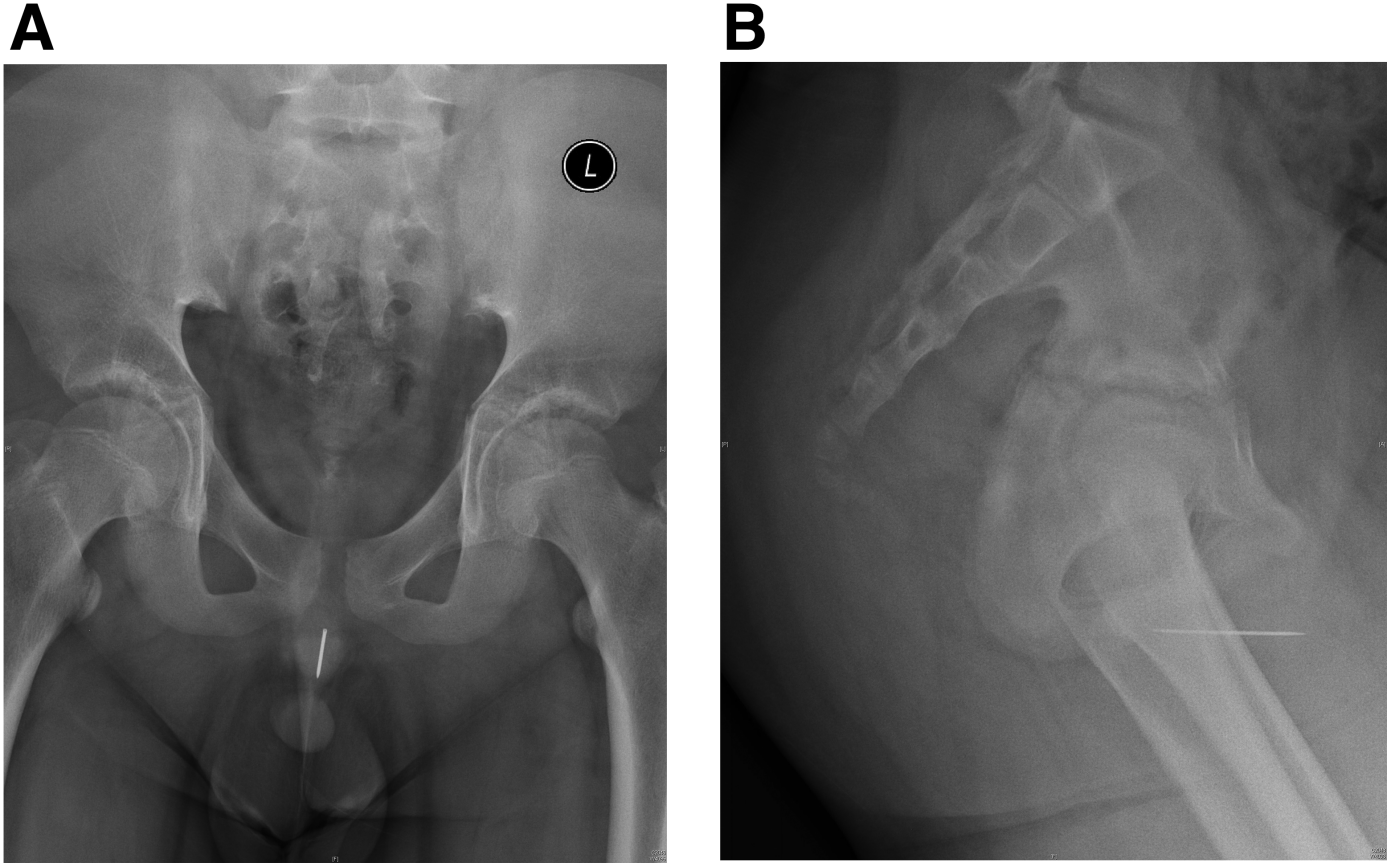
Pelvic radiography demonstrated the presence of a radiopaque, needle-like foreign body within the posterior urethra. (**A**) The anteroposterior view. (**B**) The lateral view. L, left.

An 11-year-old boy, with no past medical history, presented to our emergency department with complaints of increased frequency of micturition and dysuria, accompanied by his parents. A physical examination was unremarkable, and laboratory examinations including urinalysis and routine blood test were all within normal range. Ultrasonography showed a hyperechogenic strip without acoustic shadow in the posterior urethra. A plain x-ray of the pelvis revealed a linear radio-opaque foreign body below the symphysis pubis overlying penile soft tissue shadow, which was highly similar to a sewing needle shadow (Figure 1). On further questioning, the patient admitted to urethral self-insertion of a hand sewing needle 7 days ago due to simple curiosity. He denied any history of psychiatric disorders, domestic abuse or school bullying.

**Figure 2:**
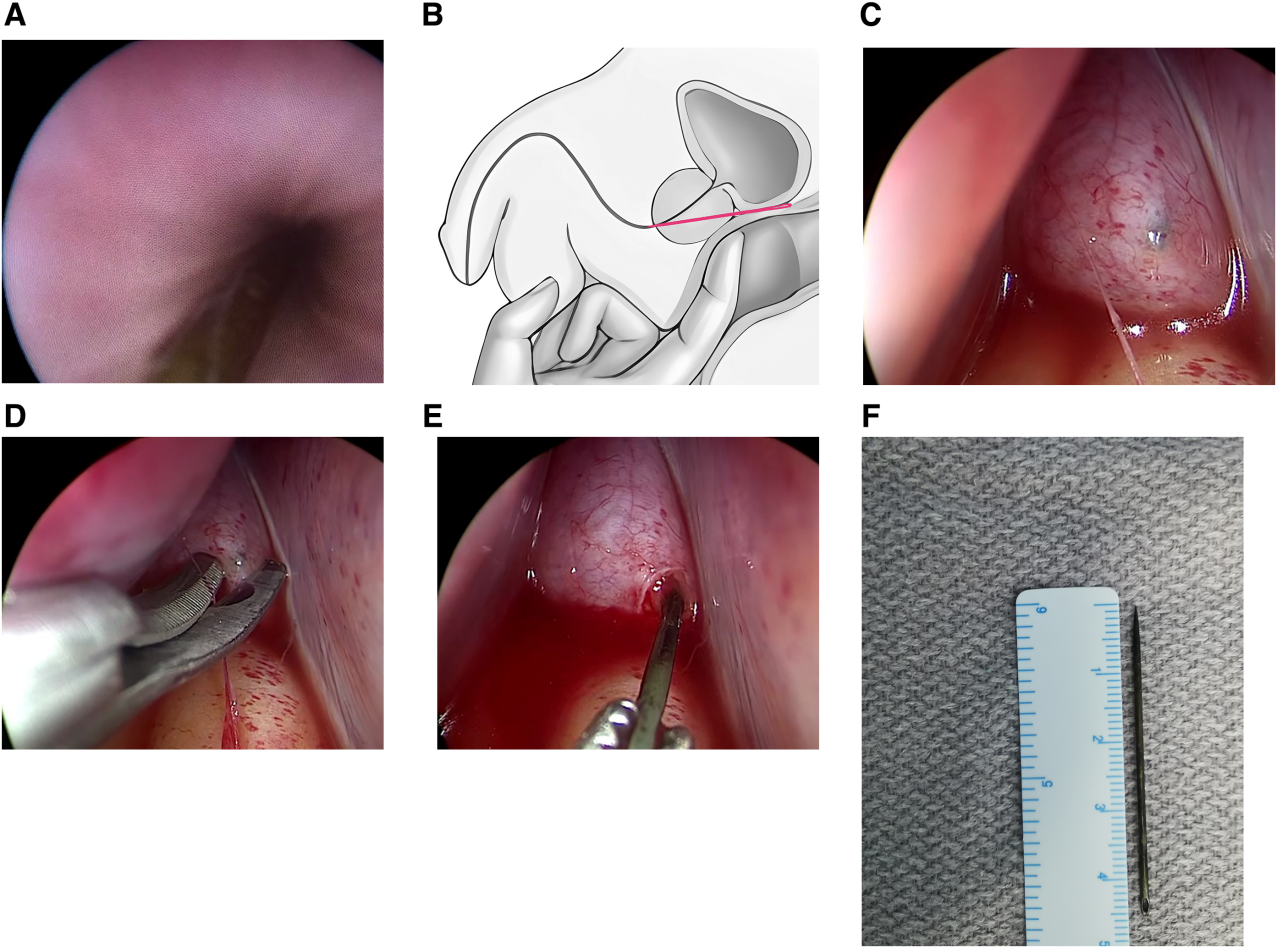
Operative images. Cystoscopy revealed a sharp sewing needle lodged in the posterior urethra mucosa (**A**). Illustration showed the needle (marked in red color) migrating into the pelvic region during a digital rectal examination (**B**). Laparoscopy revealed the needle was identified near the peritoneal reflection over the fundus of the bladder (**C**). Laparoscopy revealed the peritoneum was incised approximately 5 mm using scissors (**D**). Laparoscopy revealed the needle was grasped and extracted through a Maryland forceps (**E**). The needle was measured approximately 45 mm in length (**F**).

The patient was taken to the operating room and placed in a lithotomy position under general anesthesia. During cystoscopy, a sharp sewing needle was discovered lodged in the posterior urethra mucosa (Figure 2A). Through a 7.9 Fr pediatric cystoscope (OLYMPUS), attempts to remove the needle using an endoscopic grasping forceps were unsuccessful due to the forceps' weak biting power. Then, we conducted a digital rectal examination (DRE) to locate the needle and attempted to push it out through the perineum; however, this was also unsuccessful. Unexpectedly, the needle was pushed into the pelvic region, wedged between the prostatic urethra and the rectal ampulla (Figure 2B). Cystoscopy confirmed that the needle had penetrated through the urethra toward the anterior peritoneal reflection, with only the tip visible.

**Figure 3:**
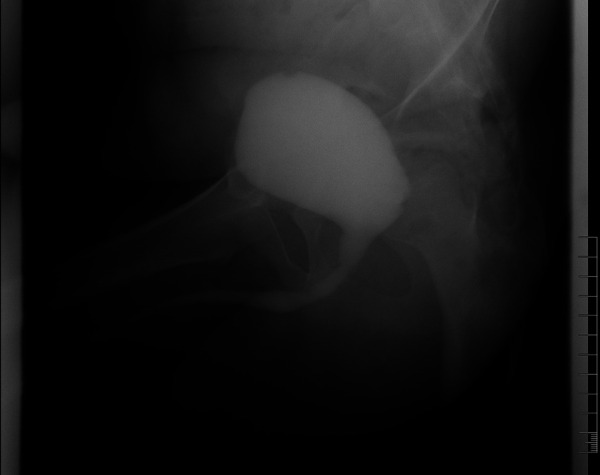
Voiding cystourethrography confirmed normal urethra and bladder of the patient.

Following a comprehensive discussion with the patient's parents and obtaining informed consent, a laparoscopic intervention was deemed necessary to extract the needle. In order to facilitate access to both the abdomen and perineum, the patient remained in a lithotomy position with a slight Trendelenburg. The surgeon and scrub nurse stood on the right side of the patient, while the camera holder was on the left side. The laparoscopic monitor was positioned at the feet end of the patient. A 10 mm port was initially inserted through an open cut-down technique at the umbilicus, which was followed by creation of pneumoperitoneum at a pressure of 12 mmHg. A 10 mm 30-degree telescope (KARL STORZ) was inserted for enhanced visualization. Two 5 mm trocars were situated on the midclavicular line at the level of the umbilicus to serve as working ports. To expose the posterior bladder wall, the bladder dome was suspended to the anterior abdominal wall using a transparietal stay suture. After careful inspection of the anterior reflection of the peritoneum over the fundus of the bladder, a needlelike black foreign body was identified, which had not penetrated through the peritoneum yet (Figure 2C). Following an approximately 5 mm incision of the peritoneum using scissors, the distal part of the needle was visualized directly (Figure 2D). A Maryland forceps was used to grasp and extract the needle, which was then removed through the right working port (Figure 2E, Supplementary Video S1). The needle was measured approximately 45 mm in length (Figure 2F). To prevent further stricture, a 14 Fr Foley catheter was inserted through the urethra. An abdominal drainage was placed in the pelvis and removed on the third day after the surgery. Oral antibiotics were prescribed for 5 days to prevent infection. The patient was discharged uneventfully on postoperative day 7. After confirming normal urethra and bladder through a voiding cystourethrogram (VCUG) on postoperative day 14 (Figure 3), the Foley catheter was removed at the outpatient clinic. Psychiatric counseling was advised for this patient, who was in good condition during an 8-week follow-up.

## 3. Discussion

Although reports of FBs in the lower urinary tract have increased in recent decades, they are still considered rare in children (3, 7). These FBs can be self-introduced, iatrogenic, migrate from adjacent organs, or arise from penetrating trauma (3, 8). Among pediatric cases, self-introduction of FBs may indicate underlying psychiatric illnesses, accidental insertions, sexual stimulation, or mere curiosity. A wide variety of self-inserted FBs include needles, magnetic beads, metallic pins, hair clips, pencils, electric wire, and more (1–10).

Affected patients typically present with cystitis, characterized by increased urinary frequency, dysuria, hematuria, and strangury. However, some patients may be asymptomatic (3). Misdiagnosis may occur if the diagnosis is based solely on clinical symptoms, particularly when patients withhold information regarding FB insertion at presentation due to feelings of shame and embarrassment (4, 5). While plain x-rays of the urinary tract can detect most FBs, abdominal and pelvic ultrasound is particularly useful for nonradio-opaque FBs (3).

The primary objective of treatment is to extract the FB with minimal or no injury to the urethra. Several methods of removal have been described, including direct extraction, endoscopic treatment, open surgery, and laparoscopic management (3, 10). The most appropriate method depends on the patient's age, the characteristics of the FB, such as its shape, size, location, and mobility within the urethra. He et al. reported a case of a 12-year-old boy with a self-inflicted sewing needle lodged at the junction of the anterior and posterior urethra. They successfully removed the needle by performing DRE and pushing it out from the perineum (7). However, during our DRE maneuvers, the needle migrated further into the pelvic cavity rather than being extruded from the perineal area. Therefore, surgeons should be cautious when performing DRE procedures to avoid iatrogenic injury caused by the inadvertent migration of sharp FBs within the urethral tract. A further problem is that the migration of sharp FBs from the urethra to surrounding areas could prevent successful removal. He et al. reported another male case with a urethral needle confirmed by cystoscopy. Despite performing intraoperative radiography, cystotomy, and episiotomy, they were unable to remove the needle and chose to leave it in the patient's body to avoid further injury (7). After confirming the distance and direction of the needle migration through cystoscopy in our case, a decision was made to conduct a pelvic laparoscopic exploration instead of a cystotomy, as the needle did not migrate into the urinary bladder. Through meticulous scrutiny of the peritoneal reflection utilizing a high-definition magnified laparoscopic view, the needle was successfully identified and extracted.

A technique was reported for the removal of complex intravesical FBs in adults, which involved introducing a laparoscopic port through a small suprapubic incision into a carbon dioxide (pneumovesicum) or saline distended bladder, guided by visualization through the cystoscope (10–12). However, this technique was unsuitable for our case. Park et al. accomplished the removal of a urethral needle from a 13-year-old boy through the direct insertion of a laparoscopic needle holder into the urethra. Rather than performing a laparoscopic surgery, they utilized a laparoscopic instrument due to its powerful biting capabilities (2). To our knowledge, our case report is the first study to document the use of pelvic laparoscopy for the removal of FBs that have migrated from the urethra to the pelvis in children and adolescents.

## 4. Conclusions

FBs in the lower urinary tract of pediatric patients have rarely been reported, and their management poses a significant challenge, particularly in male children due to the elongated and narrow urethra. Performing a DRE during removal of sharp FBs such as needles might yield unexpected outcomes. Our case demonstrates the first recorded use of laparoscopy to remove a self-inserted urethral needle that had migrated into the pelvic region, after failed attempts at endoscopic and DRE extraction. Future cases may benefit from considering laparoscopic interventions for similar circumstances.

## Data Availability

The original contributions presented in the study are included in the article/**Supplementary Material**, further inquiries can be directed to the corresponding author.

## Supplementary material

SUPPLEMENTARY VIDEO S1

Laparoscopic management of the needle.
